# Recalcitrant Foot Ulceration in a Patient With Type 1 Diabetes Mellitus

**DOI:** 10.7759/cureus.8898

**Published:** 2020-06-29

**Authors:** Stephan Vazeille, Lydia Hawker, Ramasubramanyan Chandrasekar, Upendram Srinivas-Shankar

**Affiliations:** 1 Diabetes and Endocrinology, Wirral University Teaching Hospital, Birkenhead, GBR; 2 Vascular Surgery, Wirral University Teaching Hospital, Birkenhead, GBR

**Keywords:** type i diabetes mellitus, diabetic foot ulcers management, cannula, diabetic ketoacidosis, critical care, delayed wound healing, larval therapy, surgical debridement

## Abstract

We describe the case of a middle-aged woman with type 1 diabetes mellitus who presented to the emergency department with diabetic ketoacidosis. An intravenous cannula was inserted into the veins of the dorsum of the right foot due to difficulty in obtaining intravenous access in the upper limb for managing diabetic ketoacidosis. Our patient developed edema and bullae on the dorsum of the right foot and received intravenous antibiotics for bullous cellulitis. Our patient developed ulceration on the dorsum of the right foot and over the next few months was admitted to hospital on several occasions with infected foot ulceration, which required several courses of intravenous antibiotics, larval therapy and surgical debridement of the necrotic eschar and slough. With regular review in the multidisciplinary diabetic foot clinic, the foot ulceration finally healed in eight months. This case highlights the importance of avoiding trauma in any form to the feet of people with diabetes even if aseptic techniques are taken.

## Introduction

Foot ulceration is common in people with diabetes and is associated with various complications, including infected ulceration, cellulitis, osteomyelitis, bacteremia, sepsis, gangrene, and minor and major lower limb amputations [1,2]. The prevalence of foot ulceration amongst people with diabetes is between 4% and 10%, and approximately one in four patients with diabetes develop foot ulceration during their lifetime [3]. It is important to manage foot ulceration in a timely manner as the majority of minor and major lower limb amputations are preceded by foot ulceration [4]. Various factors predispose to foot ulceration, with neuropathy, foot deformity and peripheral arterial disease being the main risk factors [5]. Often, the precipitant for foot ulceration is minor trauma to an insensate foot. Peripheral intravenous cannulation is a commonly performed minor invasive procedure for intravascular access and is associated with a range of complications, including soft tissue infection, infiltration of the infused intravenous fluid, hematoma, embolism and thrombophlebitis [6]. Here, we report the case history of a patient with type 1 diabetes mellitus who developed chronic foot ulceration secondary to intravenous cannulation on the dorsum of the right foot.

## Case presentation

**Figure 1:**
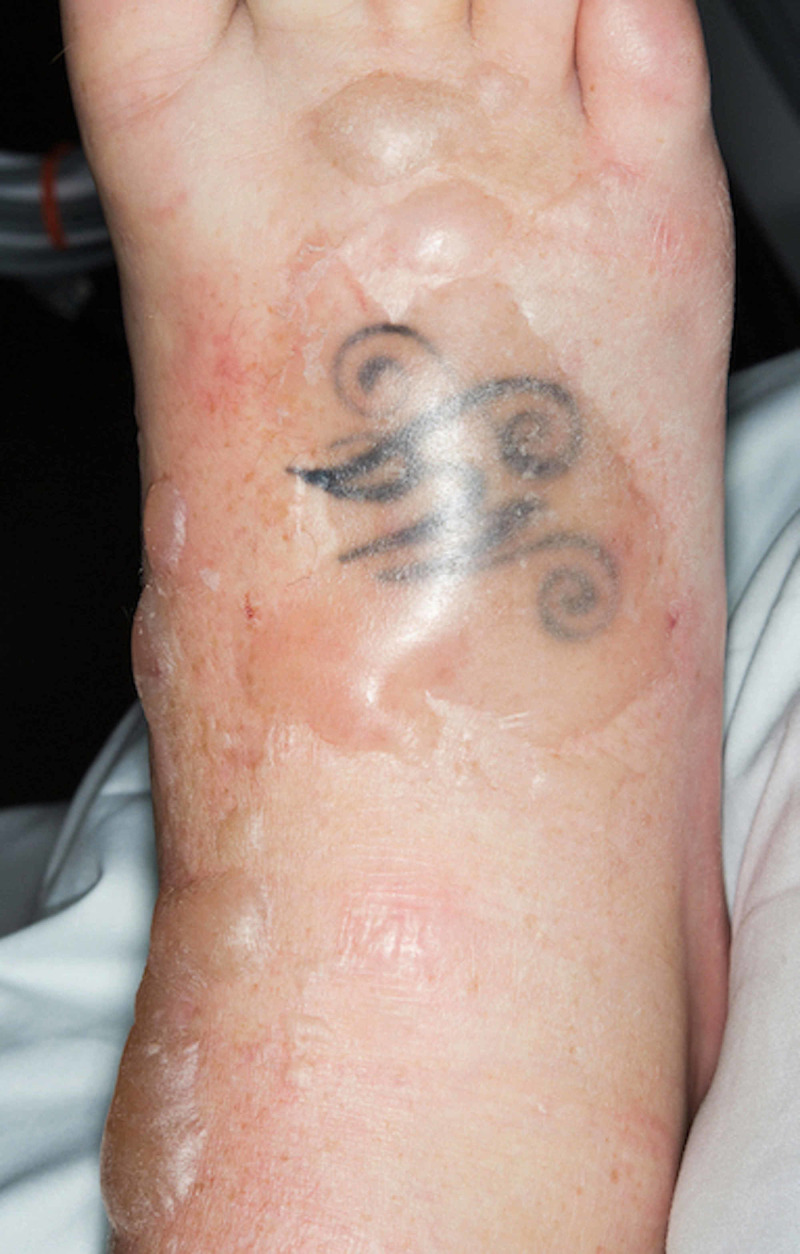
Dorsum of the right foot with bullous lesions.

A 47-year-old woman presented to the emergency department with a one-day history of vomiting and was found to be in diabetic ketoacidosis. Past medical history consisted of type 1 diabetes mellitus diagnosed at the age of 10 years, sensory neuropathy, retinopathy and hyperlipidemia. Glycemic control was poor with a glycated hemoglobin (HbA1c) of 109 mmol/mol (normal range, <42 mmol/mol), and our patient smoked 15 cigarettes a day for 30 years. Due to difficulty in obtaining intravenous access in the upper limb veins, an intravenous cannula was inserted on the dorsum of the right foot and our patient was treated for diabetic ketoacidosis. Within a few hours, our patient developed edema followed by bullous lesions on the dorsum of the right foot (Figure 1). Dorsalis pedis and posterior tibial pulsations were not palpable due to edema. Hand-held Doppler revealed biphasic dorsalis pedis and posterior tibial pulsations. Initial white blood cell count was 11.5 x 10^9^/L (normal range, 4.0-11.0 x 10^9^/L) and C-reactive protein was 69.0 mg/L (normal range, <10.0 mg/L). Our patient was treated for bullous cellulitis with intravenous flucloxacillin and discharged home.

**Figure 2:**
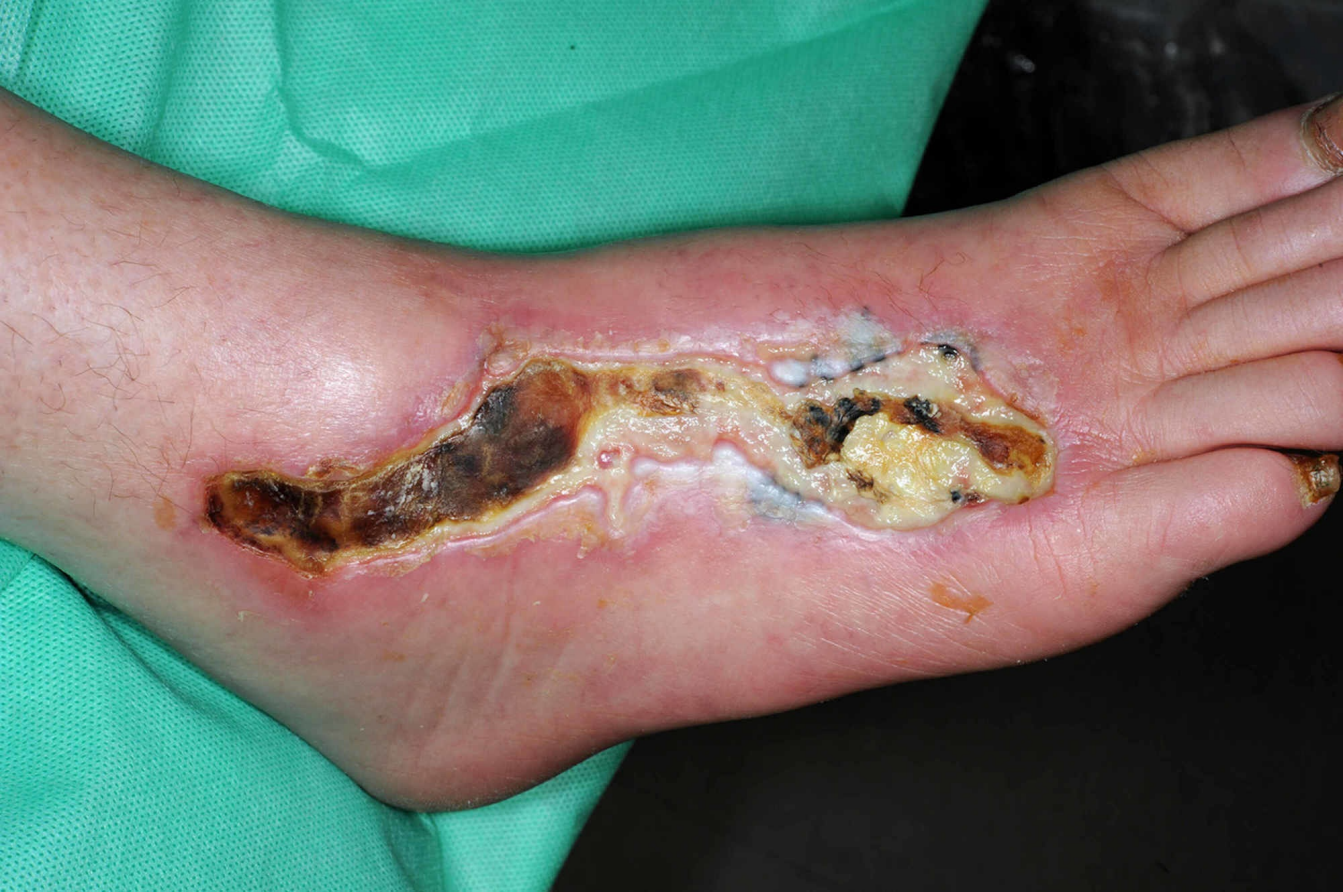
Ulceration on the dorsum of the right foot covered with a thick eschar and slough.

Nine weeks later, our patient was re-admitted to hospital with an infected foot ulceration measuring 14 x 4 cm. The ulcer was covered with a thick eschar and slough around the edges (Figure 2). X-ray of the right foot was normal. MRI revealed subcutaneous edema on the dorsum of the foot, but no osteomyelitis. Duplex ultrasound scan of the right lower limb arteries did not reveal evidence of peripheral arterial disease. Right-sided resting ankle-brachial pressure index was normal at 1.15. Catheter angiogram of the lower limb arteries revealed good perfusion of the foot. Blood cultures did not reveal bacteremia. Tissue cultures from the foot ulceration revealed various organisms including *Staphylococcus aureus*, *Staphylococcus epidermidis*, *Streptococcus pyogenes* and *Klebsiella pneumoniae* on various occasions. Based on sensitivities and following microbiological consultation, our patient received intravenous antibiotics including flucloxacillin, co-amoxiclav, teicoplanin and piperacillin/tazobactam on various occasions.

**Figure 3:**
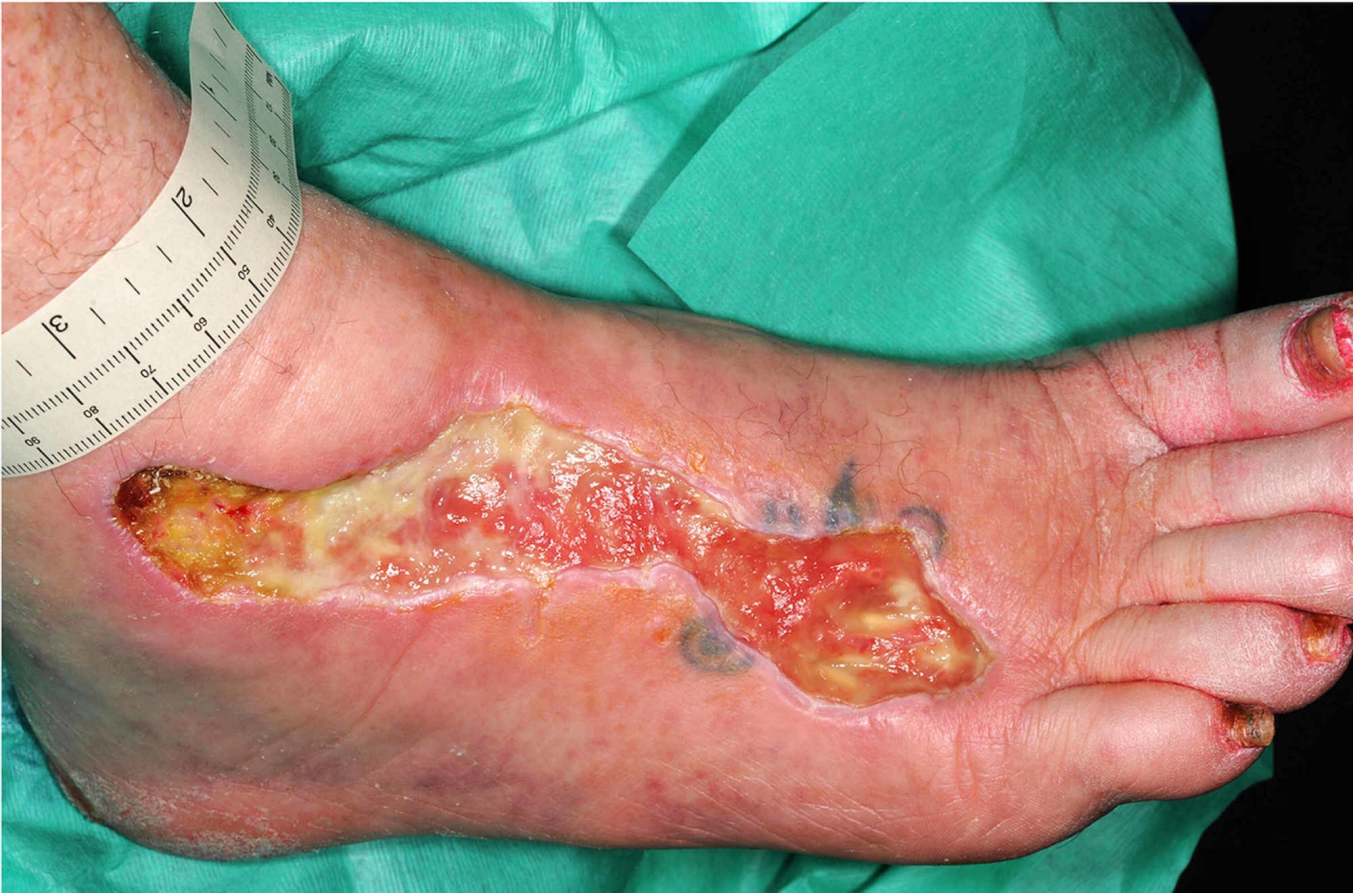
Foot ulceration after larval therapy and surgical debridement.

Fifteen weeks after initial presentation, following joint review by the diabetologist and vascular surgeon in the multidisciplinary diabetic foot clinic, our patient received larval therapy. Larval therapy involves the use of larvae of the common green bottle fly (*Lucilia sericata*) placed on the ulcers to reduce slough and debride necrotic tissue. The larvae produce proteolytic enzymes and alter wound pH which may inhibit bacterial growth-promoting agents. Further, larval secretions have growth-promoting effects, which may facilitate fibroblast migration and tissue regeneration [7]. Our patient later underwent surgical debridement on the ulceration, which reduced the amount of slough and eschar further (Figure 3). 

**Figure 4:**
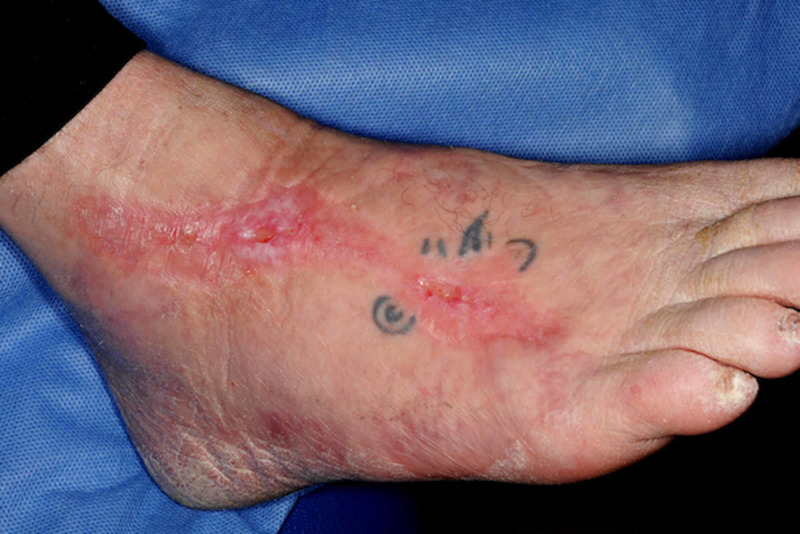
Healed foot ulceration on the dorsum of the right foot.

Our patient was referred to the orthotics team initially for temporary footwear and once the ulceration settled, for bespoke footwear. Our patient was advised to keep her foot elevated during the acute stages of the infection but did not require immobilization. With regular review in the multidisciplinary diabetic foot clinic, our patient’s foot ulceration finally healed in eight months (Figure 4). A secondary consequence of the foot ulceration was the obliteration of our patient’s tattoo on the dorsum of the right foot.

## Discussion

Often, minimal trauma in the form of abrasions from new footwear and walking bare foot are precipitants for foot ulceration in people with diabetes with various risk factors for foot ulceration [8]. In our patient, the precipitant for the foot ulceration was the insertion of an intravenous cannula into the foot. Our literature search did not reveal any reported case histories of foot ulceration in patients with diabetes caused by an intravenous cannula, although we suspect that this is not uncommon. Obtaining intravenous access in the foot should only be considered in extreme circumstances and only after other methods for securing intravenous cannulation in the upper limb veins have failed, including the application of nitroglycerin ointment, the use of a blood pressure cuff to dilate veins and the use of ultrasound-guided intravenous cannulation [9].

We believe that in addition to the trauma caused by the intravenous cannula, extravasation of the infusion fluids (normal saline and dextrose) may have occurred, precipitating bullous eruptions and ulceration in our patient. Intravenous dextrose solutions, especially concentrations above 10%, may cause tissue necrosis [10,11]. Tissue damage with bullous eruptions following extravasation of normal saline has also been reported, and clinicians need to be aware of this potential complication [12]. Bullous cellulitis, which our patient developed, is usually caused by beta-hemolytic streptococci and less commonly by *Staphylococcus aureus* and Gram-negative organisms. It is associated with local discomfort, edema, erythema and clear fluid-filled bullous lesions, as seen in our patient. The management of this condition is similar to other forms of cellulitis [13].

Our patient had microvascular disease (retinopathy and neuropathy), and it is possible that the microvascular circulation in the foot was impaired, predisposing to ulceration and delayed healing. Patients with diabetes are immunocompromised. The etiology is complex including impaired T-lymphocyte and neutrophil function [14]. *Staphylococcus aureus* is the most common causative organism of diabetic foot infections; however, foot infections may be polymicrobial with multiple Gram-negative organisms [15,16]. It is important to obtain deep wound swabs and tissue cultures to guide antibiotic treatment, as we did.

The average time for foot ulceration in people with diabetes to heal is 4-12 weeks [17]. There are several predictors of complicated diabetic foot ulceration and poor healing, including wound size and location, biofilm presence, tobacco smoking, glycemic control, associated peripheral arterial disease, chronic kidney disease and patient compliance [18,19]. In our patient, the foot ulceration took a long time to heal despite multidisciplinary care. The location and the large size of the ulceration may have contributed to slow healing as the dorsum of the foot is prone to abrasions from footwear. Factors such as peripheral neuropathy, tobacco smoking and poor glycemic control may also have contributed to delayed healing.

## Conclusions

Our case report highlights the importance of avoiding trauma in any form to the feet of people with diabetes even if aseptic techniques are taken. Further caution should be exercised when infusing intravenous dextrose as it may cause tissue necrosis and ulceration. Multidisciplinary care is important to ensure healing of foot ulceration in people with diabetes.
